# Amniotic Sludge and Prematurity: Systematic Review and Meta-analysis

**DOI:** 10.1055/s-0043-1772189

**Published:** 2023-09-08

**Authors:** Gabriel Duque Pannain, Ana Maria Gomes Pereira, Maria Luiza Toledo Leite Ferreira da Rocha, Reginaldo Guedes Coelho Lopes

**Affiliations:** 1 Departamento de Ginecologia e Obstetrícia, Instituto de Assistência Médica ao Servidor Público Estadual de São Paulo, São Paulo, SP, Brazil

**Keywords:** sludge, prematurity, amniotic fluid, *sludge*, prematuridade, líquido amniótico

## Abstract

**Objective** To perform a systematic review and meta-analysis of studies on maternal, fetal, and neonatal outcomes of women with singleton pregnancies, after spontaneous conception, and with the diagnosis of amniotic sludge before 37 weeks of gestational age.

**Data Sources** We conducted a search on the PubMed, Cochrane, Bireme, and Theses databases until June 2022.

**Selection of Studies** Using the keywords *intra-amniotic sludge* or *fluid sludge* or *echogenic particles* , we found 263 articles, 132 of which were duplicates, and 70 were discarded because they did not meet the inclusion criteria.

**Data Collection** The articles retrieved were analyzed by 2 reviewers; 61 were selected for full-text analysis, 18 were included for a qualitative analysis, and 14, for a quantitative analysis.

**Data Synthesis** Among the maternal outcomes analyzed, there was an increased risk of preterm labor (95% confidence interval [95%CI]: 1.45–2.03), premature rupture of ovular membranes (95%CI: 1.99–3.79), and clinical (95%CI: 1.41–6.19) and histological chorioamnionitis (95%CI: 1.75–3.12). Regarding the fetal outcomes, there was a significant increase in the risk of morbidity (95%CI: 1.80–3.17), mortality (95%CI: 1.14–18.57), admission to the Neonatal Intensive Care Unit (NICU; 95%CI: 1.17–1.95), and neonatal sepsis (95%CI: 2.29–7.55).

**Conclusion** The results of the present study indicate that the presence of amniotic sludge is a risk marker for preterm delivery. Despite the heterogeneity of the studies analyzed, even in patients with other risk factors for prematurity, such as short cervix and previous preterm delivery, the presence of amniotic sludge increases the risk of premature labor. Moreover, antibiotic therapy seems to be a treatment for amniotic sludge, and it may prolong pregnancy.

## Resumo

**Objetivo** Realizar revisão sistemática e metanálise de estudos que avaliaram os desfechos maternos, fetais e neonatais em gestantes de gravidez única, após concepção espontânea, e com o diagnóstico de *sludge* amniótico antes de 37 semanas de idade gestacional.

**Fontes dos dados** Realizou-se uma pesquisa nas bases de dados PubMed, Cochrane, Bireme e Teses até junho de 2022.

**Seleção dos estudos** Usando as palavras-chave *intra-amniotic sludge* ou *fluid sludge* ou *echogenic particles* , foram encontrados 263 artigos, 132 dos quais eram duplicatas, e 70 foram descartados por não corresponderem aos critérios de inclusão.

**Coleta de dados** Os artigos encontrados foram analisados por 2 revisores; 61 foram selecionados para análise de texto completo, 18 foram incluídos em uma análise qualitativa e 14, em uma análise quantitativa.

**Síntese dos dados** Entre os desfechos maternos analisados, houve aumento do risco de trabalho de parto prematuro (intervalo de confiança de 95% [IC95%]: 1.45–2.03), rotura prematura de membranas ovulares (IC95%: 1.99–3.79), e corioamnionite clínica (IC95%: 1.41–6.19) e histológica (IC95%: 1.75–3.12). Em relação aos desfechos fetais, houve aumento significativo do risco de morbidade (IC95%: 1.80–3.17), mortalidade (IC95%: 1.14–18.57), admissão em Unidade de Tratamento Intensivo (UTI) neonatal (IC95%: 1.17–1.95) e sepse neonatal (IC95%: 2.29–7.55).

**Conclusão** Os resultados do presente estudo indicam que a presença de *sludge* amniótico é um marcador de risco para parto prematuro. Apesar da heterogeneidade dos estudos analisados, até mesmo em pacientes com outros fatores de risco para prematuridade, como colo curto e trabalho de parto prematuro anterior, a presença de *sludge* amniótico aumenta o risco de trabalho de parto prematuro na gestação. Além do mais, a antibioticoterapia parece ser um tratamento para o *sludge* amniótico, e pode ser capaz de prolongar a gravidez.

## Introduction

Prematurity is one of the major problems involving obstetrics today. It is considered the main cause of neonatal mortality and morbidity, accounting for ∼ 75% of all cases, besides presenting unfavorable long-term outcomes, such as cerebral palsy and delayed neurological development. 1 2

In 1961, the World Health Organization (WHO) defined preterm birth as those occurring before 37 full weeks, or 259 days, of gestation, regardless of fetal weight. From there, it was observed that those newborns had a higher rate of complications when compared with those born after 37 weeks. 3 4 Since then, prematurity and its causes have been the subject of studies, in an attempt to prevent as much as possible its occurrence and postpone fetal birth.

Among preterm newborns, the prevalence of severe neonatal complications such as respiratory distress syndrome and necrotizing enterocolitis, which are frequent causes of admission to the Neonatal Intensive Care Unit (NICU), is 10 times higher than in those born after 37 weeks of gestation. 5

Despite the better understanding of the factors involved in premature parturition and the development of resources to inhibit preterm labor, the prevalence of prematurity in recent decades has not decreased, and it is estimated to range from 5% to 18% in the world, and from 6.4% to 15.2% in Brazil, which corresponds on average to the worldwide birth of ∼ 15 million preterm concepts. 6 7

The main cause of preterm labor is idiopathic, corresponding to 50% of the cases. Among the known causes, we highlight the presence of maternal infection, cervical insufficiency, and short cervix. 1 8 In addition, the main risk factor for preterm labor is having a history of preterm labor. 7 8

In cases of maternal infection, there is endogenous release of proinflammatory cytokines such as interleukins (ILs) 1, 2, 6, and 7, and tumoral necrosis factor alpha (TNF-α), which stimulate the increase in the production of prostaglandins and proteases in the amnion and can thus trigger uterine contractions, cervical alterations, and membrane rupture. The increase in proinflammatory cytokines is also associated with the presence of sludge, another possible marker for an amniotic inflammatory process. 9

Cervical insufficiency, in turn, is a clinical entity characterized by a cervix unable to remain closed during pregnancy. Its pathophysiology has not yet been fully understood. It is believed to be related to a structural defect in traction force at the cervical-isthmic junction that may be associated with cervical shortening secondary to tissue inflammation. This weakened cervical sphincter yields to the weight of the fetus and progresses to a late abortion or premature delivery, usually painless and rapidly evolving. This condition is often associated with the occurrence of a cervix shorter than 20 mm to 25 mm. 10 11

As well as the short cervix, which has already been established as a risk factor for preterm labor, another ultrasound finding suggested the presence of amniotic sludge as a risk factor. 12

Sludge is mentioned when the presence of hyperechogenic material floating freely within the amniotic fluid near the cervix is observed. 13 Its composition is uncertain, associations with blood clot, meconium, caseous vernix or intra-amniotic microbial biofilm have been proposed before. 14 The most accepted theory is that this material, when observed in the first half of pregnancy, is associated with an inflammatory process, whereas, in the second half, it represents a maturational process. 14 15

Its prevalence tends to increase with gestational age, and it is present in ∼ 4% of ultrasounds performed between the first and second trimesters. 15 16 Vaginal diagnosis is more accurate during this period, because, from the third trimester on, the occurrence of meconium and caseous vernix increases, which can lead to a false diagnosis and confuse the examining physician. 15 16

The present work seeks to understand whether the sludge is a risk marker for preterm labor and whether antibiotic therapy can treat it, as well as prevent prematurity.

## Materials and Methods

The present meta-analysis has been registered on the International Prospective Register of Systematic Reviews (PROSPERO) under identification CRD42022343941. The criteria used for the review were those recommended by the Preferred Reporting Items for Systematic Reviews and Meta-Analyses (PRISMA) statement. For the selection of articles, the following eligibility criteria were used:

Study design: randomized clinical trials; prospective or retrospective cohort studies; and case control studies.

Population: women with singleton pregnancies out of labor, after spontaneous conception, and with the ultrasound diagnosis of intraamniotic sludge confirmed by a medical sonographer, performed before 37 weeks, in fetuses without malformations or uteruses with anatomical alterations.

Intervention: presence of intraamniotic sludge.

Outcomes: maternal (gestational age during labor, premature rupture of ovular membranes, and clinical and histological chorioamnionitis); and neonatal (need for NICU, morbidity and mortality, and sepsis).

Review articles, case reports, articles that were not fully available, and those that did not meet the necessary criteria were excluded.

The research was conducted using the following keywords: *intra-amniotic sludge* OR *fluid sludge* OR *echogenic particles* .

Searches were performed on the PubMed, Cochrane, Bireme, Teses and Google Scholar databeses, and 263 articles were found. All of these studies were included in the electronic platform Rayyan QCRI, a web application designed to aid in the selection of articles for systematic reviews.

Initially, 132 duplicates were excluded. After reading the titles and abstracts, 70 studies were disregarded because they did not meet the inclusion criteria (41 due to the participants; 6, due to the intervention; 4, due to the outcome; and 19, because of the study design). Finally, 61 articles were selected for full-text reading.

The full-text reading stage resulted in the inclusion of 18 studies for data analysis and the exclusion of 43 studies (10, due to duplicate population; 7, due to the wrong participants; 7, because of wrong intervention; 4, due to wrong outcome; and 11, because of wrong design). The next four were excluded due to failure to meet the inclusion criteria: one was an opinion article, and three were only abstracts from conference presentations without the original published article). Studies in which the patients were in labor at the time of the evaluation were considered to have “wrong participants”, and they were excluded.

Finally, of the 18 selected articles, only 14 were eligible to be submitted to a quantitative analysis. From the remaining four, two included only pregnant women with amniotic sludge without a comparative group, and the other two evaluated the use of antibiotic therapy in all participants of the sample, without a control group.

**Fig. 1:**
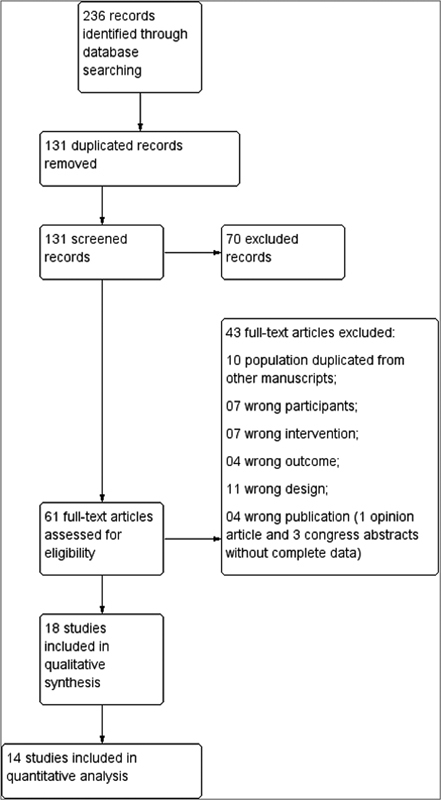
Flowchart of the selection of studies.

In all stages of the research, which can be evaluated briefly in Fig. 1 , the articles were read blindly and separately by two examiners. Disagreements were resolved after discussion with the head of the Obstetrics Department.

For data analysis, a spreadsheet was created with the following variables: study; author; country of origin; design; retrospective or prospective; duration; inclusion and exclusion criteria; initial and final number of participants; number of losses; number of participants with sludge; number of participants without sludge; gestational age at the diagnosis of sludge; maternal age; parity; history of vaginal delivery; number of abortions; history of short cervix; history of premature delivery; vaginal bleeding; smoking; cervical length in participants with sludge; cervical length in participants without sludge; performance of cerclage or use of pessary; gestational age at delivery comparing participants with sludge and without sludge; neonatal morbidity; neonatal mortality; perinatal mortality; admission to the NICU; clinical chorioamnionitis; histological chorioamnionitis; endometritis; and neonatal sepsis. The risk of bias was also independently and blindly evaluated by the authors according to the PRISMA statement and the suggestions of the Cochrane collaboration. Meta-analyses were performed when two or more studies reported the same result. The fixed-effects model was used when there was no heterogeneity (Higgins I ^2^ test < 50%), and the random-effects model, when this heterogeneity was present (Higgins I ^2^ test > 50%). The subgroup meta-analyses were performed as an attempt to reduce the bias and the unclear factors for a better understanding of the results. They were made according to the previous risk for preterm labor. Studies groups were conducted with risk, without risk and with part of the samples with risk.

## Results

After the exclusions, 14 studies were quantitatively analyzed, which comprised 546 pregnant women with an ultrasound diagnosis of intraamniotic sludge and their newborns. Chart 1 details the studies regarding the number of participants diagnosed with sludge and the control participants regarding the presence of short cervix and treatment with cerclage or pessary.

**Chart 1:** Characteristics on the population of the studies analyzed

Author, year	N sludge	N no sludge	Short cervix	Cerclage or pessary
Adanir et al., 2018 16	18	74	UR	UR
Bujold et al., 2008 17	14	75	UR	UR
Espinoza et al., 2005 18	19	65	UR	4/16 and 4/65
Gorski et al., 2010 19	60	117	Part	YES
Hatanaka et al., 2014 20	49	146	Part	UR
Himaya et al., 2011 21	16	200	Part	UR
Huang et al., 2022 22	45	251	Part	YES
Kovavisarach and Jongfuangprinya, 2019 23	72	258	Part	NO
Kusanovic et al., 2007 24	66	215	Part	28/215 and 21/66
Saade et al., 2018 25	78	579	YES	UR
Ting et al., 2012 26	5	15	YES	YES
Tsunoda et al., 2020 27	29	81	YES	6/29 and 10/81
Vaisbuch et al., 2010 28	64	45	YES	UR
Yasuda et al., 2020 29	11	43	UR	UR

Abbreviations: N, number of participants; UR, unreported.

The risk factors for the development of intraamniotic sludge, such as the occurrence of first-trimester vaginal bleeding (VB), smoking (S), and history of preterm delivery (HPTD), were also analyzed. From there, the proportion of events in the control group (non-sludge) and the experimental group (sludge) was compared, and the relative risk (RR) and its 95% confidence interval (95%CI) were as follows: VB – 1.7292 (1.2227–2.4455); S – 0.8652 (0.5087–1.4716); and HPTD – 1.2248 (0.9637–1.5566) ( Chart 2 ).

**Chart 2:** Clinical characteristics of the participants of the studies analyzed

Author, year	VB sludge	VB no sludge	S sludge	S no sludge	HPTD sludge	HPTD no sludge
Adanir et al., 2018 16	9\18	16/74	UR	UR	YES	YES
Bujold et al., 2008 17	UR	UR	UR	UR	4/14	20/75
Espinoza et al., 2005 18	6\19	3\65	UR	UR	3\19	21/65
Gorski et al., 2010 19	10\60	19\117	5\60	8\117	18/60	34/117
Hatanaka et al., 2014 20	UR	UR	UR	UR	UR	UR
Himaya et al., 2011 21	UR	UR	UR	UR	3\16	13/200
Huang et al., 2022 22	UR	UR	UR	UR	7\45	40/251
Kovavisarach and Jongfuangprinya, 2019 23	UR	UR	UR	UR	UR	UR
Kusanovic et al., 2007 24	16/66	25/215	10/57	43/192	21/66	74/215
Saade et al., 2018 25	UR	UR	UR	UR	UR	UR
Ting et al., 2012 26	UR	UR	UR	UR	UR	UR
Tsunoda et al., 2020 27	3\29	2\81	0/29	0/81	7\29	10\81
Vaisbuch et al., 2010 28	UR	UR	UR	UR	UR	UR
Yasuda et al., 2020 29	UR	UR	1\11	0\43	3/11	5/43

Abbreviations: HPTD, history of preterm delivery; S, smoking; UR, unreported; VB, first-trimester vaginal bleeding.

Despite their exclusion from the quantitative analysis, the four studies on were submitted to a qualitative analysis, and are summarized in chart 3 .

**Chart 3:** Characteristics of the studies involving antibiotic therapy

Author, year	N sludge * +* ATB	N no sludge	Antibiotic scheme	Outcome
Cuff et al., 2020 30	46	51	Azithromycin OA + moxifloxacin OA	There was no reduction in the incidence of PTD
Hatanaka et al., 2019 31	64	0	Clindamycin OA + cephalexin OA, clindamycin IV + cefazolin IV	Reduction in the incidence of PTD
Hu Jin et al., 2021 32	58	0	Ceftriaxone IV + clarithromycin OA + metronidazole IV	Reduction in the incidence of PTD and neonatal complications
Pustotina, 2020 33	14	47	Clindamycin VA, butoconazole VA, cefoperazone + sulbactam IV, amoxicillin + clavulonate OA	Reduction in the incidence of PTD, intrauterine and amniotic infection

Abbreviations: IV, intravenous; N, number of participants; OA, oral administration; PTD, preterm delivery VA, vaginal administration.

**Fig. 2:**
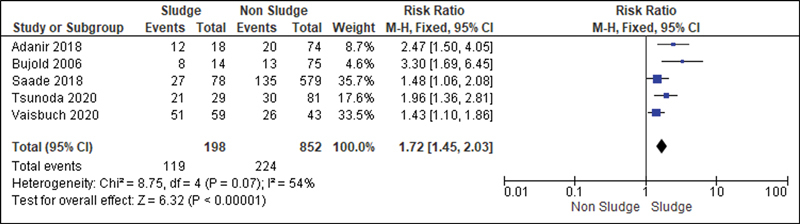
Studies in which all patients had risk factors for preterm labor.

**Fig. 3:**
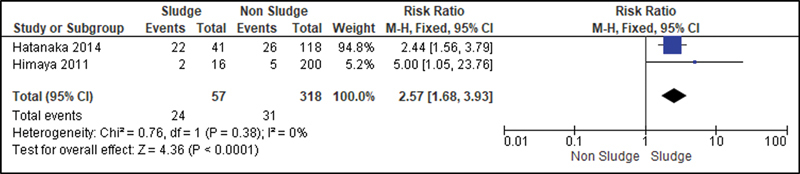
Studies in which some patients had risk factors for preterm labor and others did not.

**Fig. 4:**
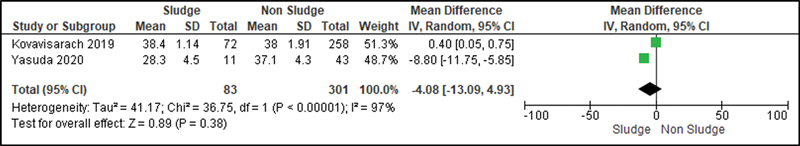
Studies in which the patients were not at risk for detected preterm labor.

**Fig. 5:**
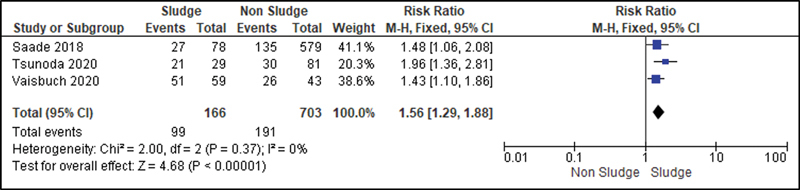
Subgroup analysis of patients with short cervix.

All studies were analyzed to establish a relationship between the risk of developing sludge and preterm delivery (before 37 weeks of gestation). Then, we observed that the Higgins I ^2^ test was of 97%. To decrease such heterogeneity and improve the statistical analysis, we divided the patients into three subgroups according to the presence or not of risk factors for premature labor, such as short cervix, HPTD or cervical insufficiency. Thus, we analyzed studies in which all patients had risk factors for preterm labor ( Fig. 2 ); studies in which some patients had these risk factors and others did not ( Fig. 3 ); studies in which the patients were not at risk for detected preterm labor ( Fig. 4 ). In the group of high-risk patients, we also analyzed a subgroup in which all participants had a short cervix ( Fig. 5 ).

**Fig. 6:**
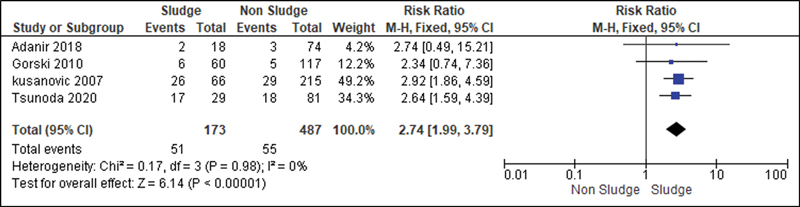
Risk of premature rupture of ovular membranes according to the presence of sludge.

**Fig. 7:**
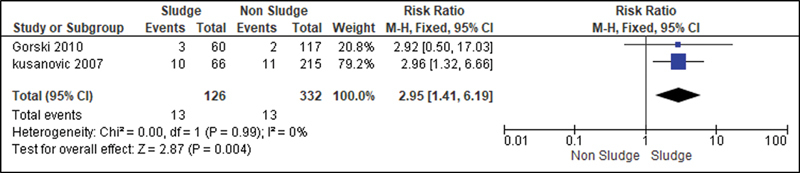
Risk of clinical chorioamnionitis according to the presence of sludge.

**Fig. 8:**
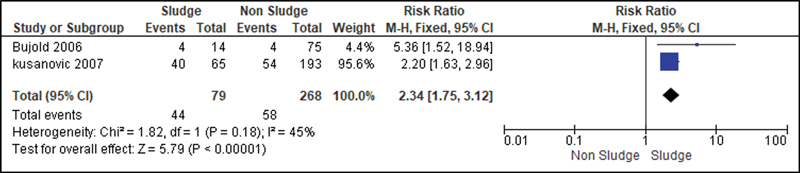
Risk of histological chorioamnionitis according to the presence of sludge.

**Fig. 9:**
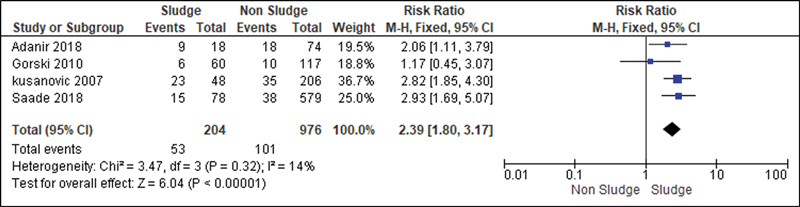
Risk of neonatal morbidity according to the presence of sludge.

**Fig. 10:**
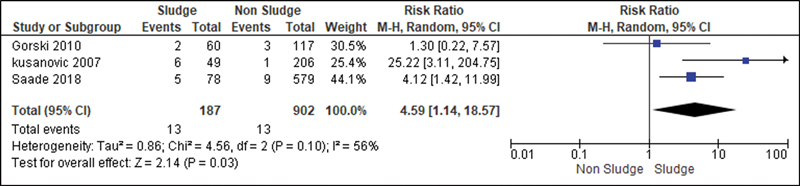
Risk of neonatal mortality according to the presence of sludge.

**Fig. 11:**
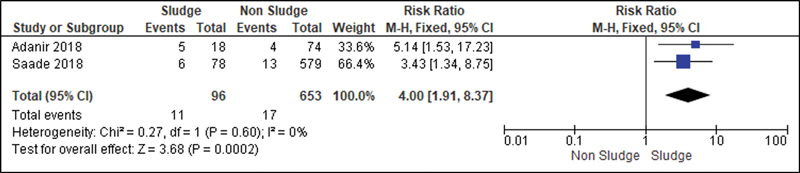
Risk of perinatal mortality according to presence of sludge.

**Fig. 12:**
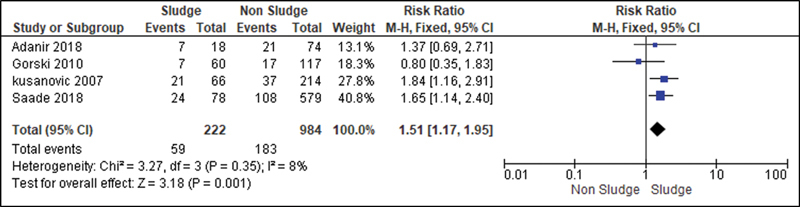
Risk of admission to the NICU according to presence of sludge.

**Fig. 13:**
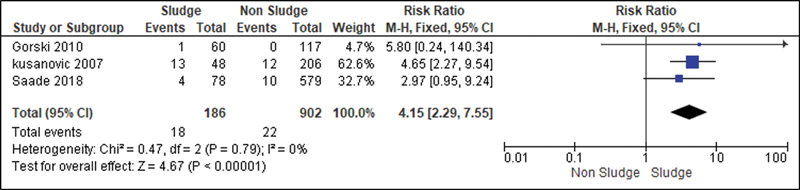
Risk of neonatal sepsis according to presence of sludge.

For the analysis of the secondary outcomes, since there was no interference regarding the presence of risk factors, there was no need for subdivision. Thus, the risk caused by the sludge was analyzed for the following variables: premature rupture of ovular membranes in preterm delivery ( Fig. 6 ); clinical chorioamnionitis ( Fig. 7 ); histological chorioamnionitis ( Fig. 8 ); neonatal morbidity ( Fig. 9 ); neonatal mortality ( Fig. 10 ); perinatal mortality ( Fig. 11 ); admission to the NICU ( Fig. 12 ); and neonatal sepsis ( Fig. 13 ).

## Discussion

Sludge is a controversial subject due to the scarcity of studies and the heterogeneity of the diagnostic criteria. To investigate its nature, several studies have assessed the presence of risk factors, such as vaginal bleeding during the first trimester, S and HPTD. Of those, statistical significance was observed only for vaginal bleeding during the first trimester, whose RR was of 1.7292 (95%CI: 1.2227–2.4455). Rust et al. 34 proposed that intraamniotic sludge is composed of blood clot secondary to vaginal bleeding, which, in the first trimester of pregnancy, is a risk factor for sludge, and, consequently, for premature labor, as described in the present study. In addition, the blood clot can be a means for bacterial growth and feeding, favoring the formation of local biofilms. 35

Regarding S and HPTD, no conclusion was deemed possible due to the heterogeneity of the published studies. According to Iams et al., 36 S can alter the physiological vaginal flora. This vaginal dysbiosis is a well-established risk factor for premature labor and premature rupture of ovular membranes, since the main route of intraamniotic infection is the microbial ascension of the lower genital tract. 35 36

To reduce the heterogeneity of studies in the risk analysis of preterm delivery, the presence of sludge in different groups was evaluated. Then, we noticed that the presence of sludge was an important risk marker for premature labor in studies involving only high-risk patients (RR: 1.72; 95%CI: 1.45–2.03), when the population was heterogeneous (RR: 2.57 (95%CI: 1.68–3.93), and in patients with short cervix (RR: 1.56; 95%CI: 1.29–1.88).

However, caution should be exercised in the analysis of these results among low-risk patients. Yasuda et al 29 showed that low-risk patients also present a higher risk of preterm labor when they have intraamniotic sludge, unlike the study by Kovavisarach and Jongfuangprinya. 23 This research, conducted in 2019 in Thailand, is the only one in which the presence of intraamniotic sludge was only analyzed by one operator, and it was a study designed to determine the prevalence of sludge in patients at low risk for premature labor. In addition to the possibility that this study design may have hindered statistical analysis, the choice of low-risk patients for preterm labor may have excluded patients with risk factors for sludge, skewing their analysis.

Although amniotic fluid is not considered a sterile environment, the presence of intraamniotic sludge was associated with the risk of developing both clinical (RR: 2.95; 95%CI: 1.41–6.19) and histological chorioamnionitis (RR: 2.34; 95%CI: 1.7–3.12), which strengthens the hypothesis that sludge is a marker of microbial infection, which is the main known cause of premature labor. 37

These facts reiterate the hypothesis that sludge is an indicator of microbial invasion within the amniotic cavity, which would explain its relationship to clinical and histological chorioamnionitis, premature labor, and neonatal sepsis.

In the present study we have also attempted to evaluate the relationship between sludge and the use of antibiotic therapy. However, the literature on this subject is scarce, making the meta-analysis impracticable. Pustotina 33 conducted a prospective study with 29 patients with sludge who were submitted to several antimicrobial regimens, such as clindamycin 100 mg vaginally for 3 days, a single intravaginal dose of 5g of butoconazole 2% cream, cefoperazone in combination with sulbactam 2 g intravenous twice a day for 5 days, and amoxicillin in combination with clavulanate 1 g orally twice a day for 5 days. In addition, all patients received oral treatment with probiotics as proposed by De La Cochetière et al. 38 not to alter the vaginal microbiota. Even if not following a pattern, the study by Pustotina 33 showed that antibiotic therapy reduced the incidence of premature labor and intrauterine and intraamniotic infection. However, there was no group that did not receive antibiotic therapy. So, to avoid confusion, this study 33 was excluded from the present meta-analysis, since there is no suggestion in the literature that this therapy may influence the outcomes related to sludge, whether positive or not.

In 2021, Jin et al. 32 conducted a retrospective cohort study in which 58 patients with uterine contraction with sludge received intravenous ceftriaxone once a day, clarithromycin 500 mg orally every 12 hours, and metronidazole 500 mg intravenously every 8 hours for up to 4 weeks. These patients were followed up until delivery, and the authors 32 observed that, in patients in which the sludge did not disappear after antibiotic therapy, there was a higher rate of premature labor and neonatal complications. They concluded that antibiotics in some patients with uterine contractions were able to eradicate intraamniotic sludge, and, in comparison with patients in whom the sludge remained, they presented a higher rate of premature labor and unfavorable neonatal outcomes.

In 2019, Hatanaka et al. 31 published a retrospective cohort of 86 patients with sludge, in which low-risk patients received clindamycin 300 mg orally every 6 hours and cephalexin 500 mg orally every 6 hours for 7 days, and high-risk patients received intravenous clindamycin 600 mg every 8 hours and cefazoline 1 g every 8 hours for 5 days, followed by another 5 days of oral treatment. This study 31 presented results similar to those of Pustotina 33 and Jin et al, 32 with the authors concluding that antibiotic therapy reduced the incidence of premature labor.

On the other hand, Cuff et al. 30 conducted a retrospective cohort study on patients diagnosed with sludge who received azithromycin 500 mg orally on day 1 followed by 250 mg orally on days 2 to 5, or moxifloxacin 400 mg orally for 5 days, and compared them with patients who did not receive antibiotic therapy. The authors 30 did not observe a reduction in the rates of preterm labor, and clinical or histological chorioamnionitis, which may suggest that either the dosage was inadequate, or such antibiotics are not effective in the treatment of sludge. This would explain why the results of this study 30 are different from those of the others previously described, which used antibiotics such as clindamycin and β-lactams and showed a reduction in the incidence of premature labor.

In view of what was exposed, one of the greatest limitations of the present study was the heterogeneity of the studies analyzed, which did not enable a meta-analysis of the articles on antibiotic therapy in the treatment of sludge, since Pustotina 33 administered antibiotic therapy to all patients, without a control group, Hatanaka et al. 31 and Cuff et al. 30 only analyzed patients with sludge, with no control group, and Jin et al 32 assessed patients with uterine contractions, unlike all other studies analyzed.

Another relevant aspect is that retrospective studies using ultrasound criteria, like all those herein evaluated, should be analyzed with caution, since they study a static image that may indicate a false diagnosis and skew the entire result. 22 Moreover, there is still no consensus on the diagnosis of sludge, which is often confused with vernix and meconium, especially when ultrasound is performed later during pregnancy. 22

Furthermore, the studies tend to publish only positive results, which may explain why each study evaluated a different outcome. This may also explain the difficulty in finding outcomes that were evaluated by more than two or three authors, as well as elucidate why the current study has so many statistically significant results.

Nevertheless, we could analyze and conclude that intraamniotic sludge is related to unfavorable neonatal outcomes. From the moment that sludge is related to preterm labor, it becomes an indirect risk factor for peri- and neonatal morbidity and mortality, since the lower the gestational age in childbirth, the higher the risk of severe complications, admission to the NICU, and neonatal sepsis, as demonstrated in the present study. 16 19 24 26

Even with the limitations of the present study, it is worth considering intraamniotic sludge as an important risk marker for premature labor, including in high-risk women, such as those with short cervix. This finding at the beginning of pregnancy should justify the referral of pregnant women to high-risk prenatal care, a more careful analysis to screen for prematurity and consideration regarding the administration of prophylactic corticotherapy in the third trimester.

## Conclusion

Despite the heterogeneity of the studies reviewed, we concluded that sludge is a risk marker for preterm labor, as well as an independent risk factor for high-risk patients, such as those with short cervix. It also appears to be a marker of intraamniotic infection, and it is related to first-trimester vaginal bleeding. Nevertheless, further studies are necessary to investigate the efficacy of antibiotic therapy in the treatment and prevention of prematurity.
